# Recommendations on neurologic, cognitive, and psychiatric manifestations in patients with Sjögren’s disease by the Brazilian Society of Rheumatology

**DOI:** 10.1186/s42358-025-00438-7

**Published:** 2025-02-11

**Authors:** Fabiola Reis de Oliveira, Simone Appenzeller, Sandra Gofinet Pasoto, Marilena Leal Mesquita Silvestre Fernandes, Maria Lucia Lemos Lopes, Sonia Cristina de Magalhães Souza Fialho, Aysa Cesar Pinheiro, Laura Caldas dos Santos, Valeria Valim, Erica Vieira Serrano, Sandra Lucia Euzébio Ribeiro, Tatiana Nayara Libório‑Kimura, Danielle Christinne Soares do Egypto, Diego Ustárroz Cantali, Juliana D’Agostino Gennari, Samira Tatiyama Miyamoto, Karina Gatz Capobianco, Alisson Aliel Vigano Pugliesi, Vinicius Tassoni Civile, Ana Carolina Pereira Nunes Pinto, César Ramos Rocha-Filho, Aline Pereira da Rocha, Virginia Fernandes Moça Trevisani

**Affiliations:** 1 Hospital das Clínicas da Faculdade de Medicina de Ribeirão Preto da Universidade de São Paulo (HCFMRP-USP), Av. Bandeirantes 3900, Vila Monte Alegre, Ribeirão Preto, SP CEP: 14049-900 Brazil; 2 Departamento de Ortopedia, Reumatologia e Traumatologia, Faculdade de Ciências Médicas, Universidade Estadual de Campinas, R. Tessália Vieira de Camargo, 126 - Cidade Universitária, Campinas, SP CEP: 13083-887 Brazil; 3 Disciplina de Reumatologia, Hospital das Clínicas HCFMUSP, Faculdade de Medicina, Universidade de São Paulo, R. Dr. Ovídio Pires de Campos, 225 - Cerqueira César, São Paulo, SP CEP: 05403-010 Brazil; 4 Disciplina de Reumatologia, Hospital Federal dos Servidores do Estado do Rio de Janeiro, R. Sacadura Cabral, 178, Saúde, Rio de Janeiro, RJ CEP: 20221-903 Brazil; 5 Disciplina de Reumatologia, Departamento de Clínica Médica, Universidade Federal de Ciências da Saúde de Porto Alegre (UFCSPA), R. Sarmento Leite, 245 - Centro Histórico de Porto Alegre, Porto Alegre, RS CEP: 90050-170 Brazil; 6 Irmandade da Santa Casa de Misericórdia de Porto Alegre, Porto Alegre, Brazil; 7 Departamento de Reumatologia, Universidade Federal de Pernambuco, Av. Prof. Moraes Rego, 1235, Cidade Universitária, Recife, PE CEP: 50670-901 Brazil; 8 Departamento de Oftalmologia, Escola Paulista de Medicina, Universidade Federal de São Paulo (EPM-UNIFESP), Rua Botucatu, 820, Vila Clementino, São Paulo, SP CEP: 04023-062 Brazil; 9 Departamento de Clínica Médica, Serviço de Reumatologia, Universidade Federal do Espírito Santo, Pós Graduação em Saúde Coletiva, Hospital Universitário Cassiano Antônio de Moraes, Av. Marechal Campos, 1468, Maruípe, Vitória, ES CEP: 29075-910 Brazil; 10 Hospital Universitário Cassiano Antônio de Moraes, Vitória, Brazil; 11 Departamento de Clínica Médica, Serviço de Reumatologia, Universidade Federal do Espírito Santo, Hospital Universitário Cassiano Antônio de Moraes, Av. Marechal Campos, 1468, Maruípe, Vitória, ES CEP: 29075-910 Brazil; 12 Disciplina de Reumatologia, Faculdade de Medicina, Universidade Federal do Amazonas, Rua Afonso Pena, 1053, Manaus, AM CEP: 69020-160 Brazil; 13 Disciplina de Reumatologia, Departamento de Medicina Interna, Centro de Ciências Médicas, Universidade Federal da Paraíba (UFPB), Campus I - Lot. Cidade Universitária, Paraíba, PB CEP: 58051-900 Brazil; 14 Serviço de Reumatologia, Hospital São Lucas da Pontifícia Universidade Católica do Rio Grande de Sul (PUCRS), Av. Ipiranga, 6690 ‑ Jardim Botânico, Porto Alegre, RS CEP: 90610‑000 Brazil; 15 Serviço de Reumatologia da Santa Casa de São Paulo, R. Dr. Cesário Mota Júnior, 112, Vila Buarque, São Paulo, SP CEP: 01221-020 Brazil; 16 Departamento de Educação Integrada em Saúde, Universidade Federal do Espírito Santo, Av. Marechal Campos, 1468, Maruípe, Vitória, ES CEP: 29040-090 Brazil; 17 Hospital Moinhos de Vento, R. Ramiro Barcelos, 910, Bairro Floresta, Porto Alegre, RS Brazil; 18 Disciplina de Medicina de Urgência e Medicina Baseada em Evidências, Escola Paulista de Medicina, Universidade Federal de São Paulo, Rua Botucatu, 740, Vila Clementino, São Paulo, SP, CEP: 04023-062 Brazil; 19 Centro Cochrane Iberoamericano, C/Sant Antoni M. Claret 167 Building 18, ground floor, Barcelona, 08025 Spain; 20 Disciplina de Reumatologia, Universidade de Santo Amaro, Rua Enéas Siqueira Neto, Jardim das Imbuias, São Paulo, SP CEP: 04829-300 Brazil

**Keywords:** Neurological manifestations, Psychiatric disease, Cognitive impairments, Peripheral nervous system, Central nervous system, Sjögren’s syndrome, Sjögren’s disease.

## Abstract

**Background:**

Neurological and psychiatric manifestations occur in patients with primary Sjogren’s disease (SjD) with a wide-ranging clinical presentation, affecting quality of life, social participation, and prognosis. Despite this, neither central nor peripheral neurological symptoms are systematically evaluated in the context of autoimmunity or identified as manifestations of SjD. The EULAR Sjogren’s Syndrome Disease Activity Index (ESSDAI) covers only part of them in the neurological domain.

**Methods:**

We performed a systematic review of the diagnosis and prevalence of central, peripheral, and autonomic nervous system manifestations in primary SjD, following the recommendations proposed by the Cochrane Collaboration Handbook. Observational studies were included when their main issue was the diagnosis and the prevalence of the manifestations individually. We employed a generalized linear mixed model (GLMM) method with a random-effects model, and the results were computed using logit transformation, implemented through the ‘meta’ and ‘metafor’ packages in the R software (version 3.6.1). To present these recommendations, agreement among experts was investigated using the Delphi method in in-person meetings.

**Results:**

We propose ten recommendations regarding the investigation and management of neurological involvement in SjD that had 100% agreement among participants.

**Conclusion:**

These recommendations add to the literature on the clinical care of patients with SjD.

**Supplementary Information:**

The online version contains supplementary material available at 10.1186/s42358-025-00438-7.

## Introduction

Sjögren’s disease (SjD) is a chronic, immune-mediated, inflammatory disease characterized by lymphocytic infiltration in tissues, exocrine dysfunction, and production of autoantibodies, typically anti-Ro/SS-A and anti-La/SS-B [1]. The diversity of extra glandular manifestations combined with dry symptoms generates a vast phenotypic polymorphism characteristic of the disease [2, 3]. In this context, the addition of neurological, psychiatric, and cognitive impairments affects quality of life (QOL) and social participation [2, 4].

Several neurological manifestations are already within the disease activity assessment tool, the EULAR Sjögren’s Syndrome Disease Activity Index (ESSDAI), such as vasculitis and demyelinating conditions of the central nervous system (CNS), cranial nerve, and peripheral nervous system (PNS) involvement [5, 6]. However, neurological and psychiatric disorders have not been evaluated systematically in individuals with SjD, nor are they yet clearly attributed to disease mechanisms [7]. The frequency of neurological impairment differs depending on the criteria for SjD classification, the recruited population (from Internal Medicine, Rheumatology, or Neurology centers), and the methods of measuring complaints [8, 9]. The heterogeneity of the diagnostic tools—clinical, serological, electromyographic, imaging, and histological analyses—contributes to the inconsistencies. Furthermore, the assortment of psychosomatic complaints, pain, and fatigue among SjD patients, the lack of a multidisciplinary team, and limited access to complementary tests increase misdiagnosis [10–12]. Thus, if headache, cognitive dysfunction, and affective disorders are primarily attributed to SjD, the frequency can reach 68% [13, 14]. Most authors, however, strictly consider the complaints caused by organic lesions of SjD, resulting in a lower prevalence and dominance of PNS involvement [15–23].

A Brazilian cohort described frequencies of 5% for CNS and 11.6% for PNS involvement in 198 patients with primary SjD. Only manifestations included in the neurological domain of ESSDAI were reported, with no mention of small fiber neuropathies, dysautonomia, or cognitive and psychiatric disorders [24]. The frequency was comparable to those (2.3–16%) reported in the previous series worldwide [15–17, 19–21, 25–28].

Pathophysiology bases of neurological, psychiatric, and cognitive disturbances resulting from SjD remain unclear. There are no well-defined criteria for diagnosis, investigation, or management. The treatment of each manifestation in SjD follows the guidelines for the primary forms. In addition, controversies regarding the association of neuropsychiatric involvement with disease phenotypes, disease activity, and extra glandular manifestations persist unsolved. Finding common molecular signatures combined with clinical parameters may help identify distinct patterns of immune dysregulation and improve diagnostic and therapeutic management in the future [29, 30].

The main objective of the present study is to promote a systematic review of psychiatric and nervous system involvement in patients with SjD and propose recommendations for diagnostic approaches.

## Methods

A systematic review was conducted on the diagnosis and prevalence of CNS, PNS, and mood dysfunctions in patients with primary Sjögren’s disease, following the recommendations proposed by the Cochrane Collaboration Handbook [31]. Questions regarding the diagnosis and prevalence of neurological and psychiatric manifestations in SjD guided a search in the Cochrane Central, MEDLINE, Embase, and LILACS databases (Appendix 1). The initial strategy had no restrictions on language or publication date. Observational studies, predominantly retrospective studies, focusing on the diagnosis and prevalence of each manifestation were selected. We subsequently excluded case reports and studies not published in English. A complementary manual search was also conducted in the reference lists of potentially eligible studies to identify any evidence not retrieved by the initial database search. Since overlap with other rheumatic diseases, such as systemic lupus erythematosus and rheumatoid arthritis, can significantly modify the frequency and the phenotype of neurological manifestations, studies containing secondary SjD were also excluded. The diagnosis of SjD was established preferably according to the 2002, 2012, or 2016 classification criteria [32–34]. Methodological quality was performed by Risk of bias using the Joanna Briggs Institute prevalence critical Appraisal Tool and Quality Assessment of Diagnostic Accuracy Studies-QUADAS-2 [35, 36] (Appendix 2). Outcomes were the number and percentage of patients with the clinical manifestation for prevalence studies and sensitivity and specificity for accuracy studies. To estimate an overall proportion and present pooled results along with their respective 95% confidence intervals (CI), we employed a generalized linear mixed model (GLMM) method with a random-effects model. The results were computed using logit transformation, implemented through the R software’s ‘meta’ and ‘metafor’ packages (version 3.6.1). The reviewed literature supported the recommendations proposed by the Sjögren’s Disease Committee of the Brazilian Society of Rheumatology. A hybrid meeting with the participation of 19 SjD specialists (15 rheumatologists, one ophthalmologist, one neurologist, and two allied health professionals) defined the recommendation. After that, the agreement among experts was investigated using the Delphi method.

In compliance with ethical norms, this article is based on previously conducted studies and did not require the approval of an ethics committee.

## Results

**Fig. 1:**
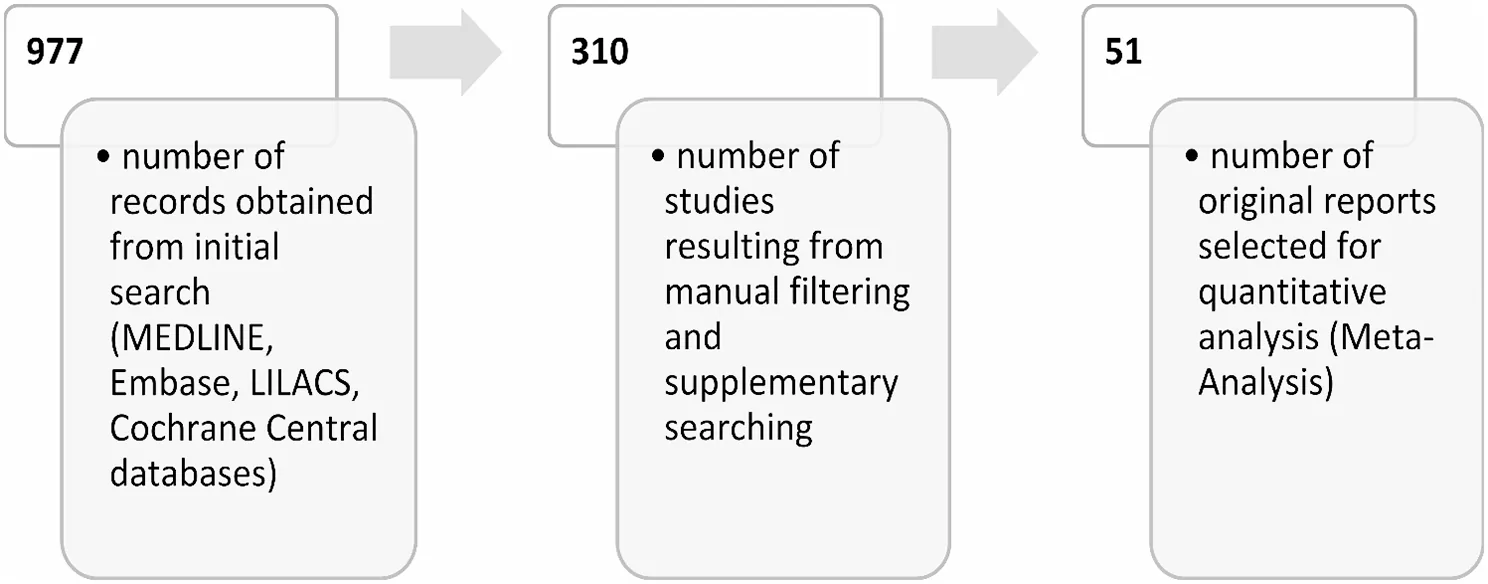
Flowchart of the studies regarding the diagnosis and prevalence of neurological and psychiatric manifestations in Sjogren’s disease selected for meta-analysis

**Fig. 2:**
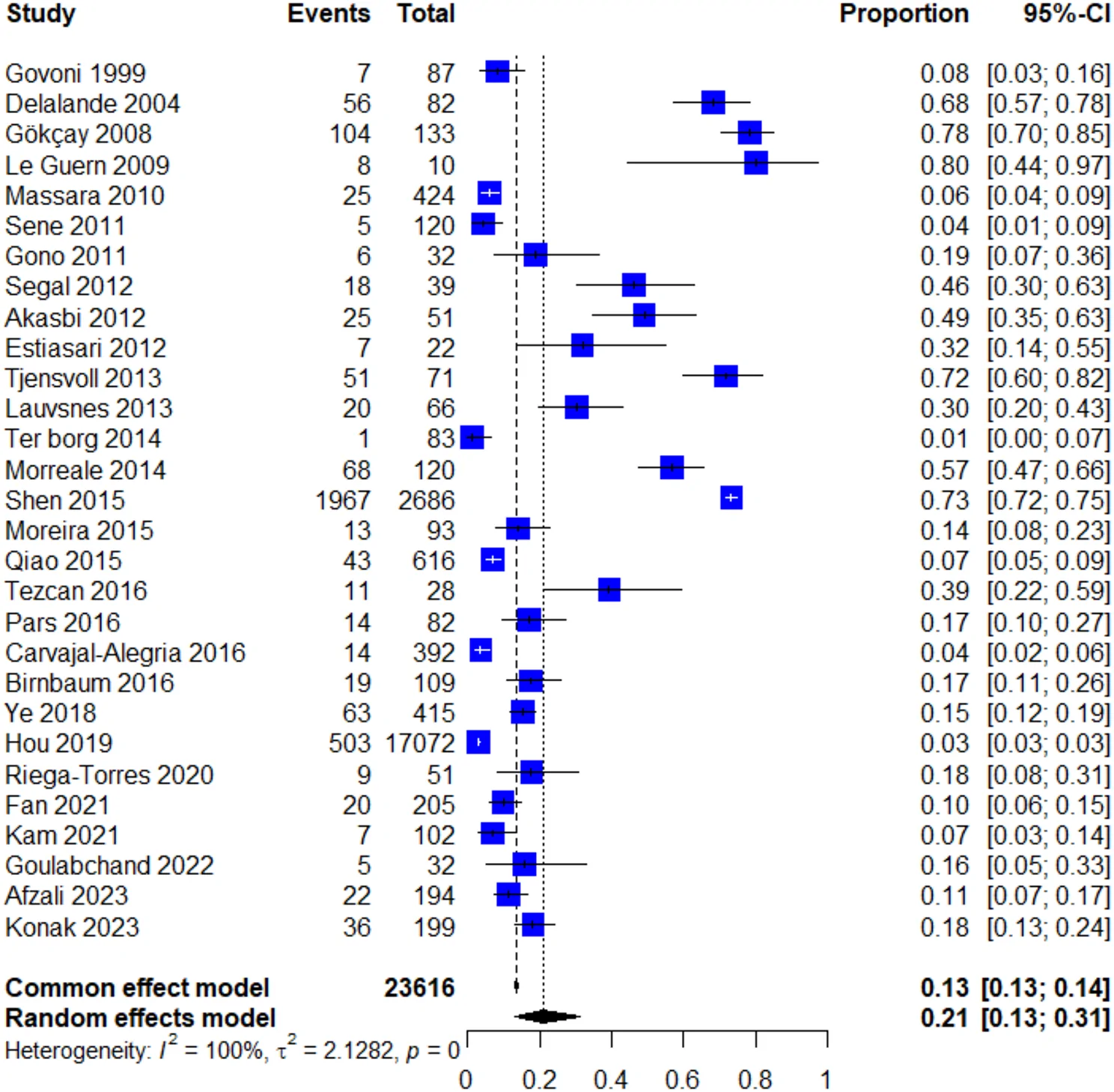
Meta-analysis of the peripheric nervous system manifestations in patients with Sjogren’s disease

**Fig. 3:**
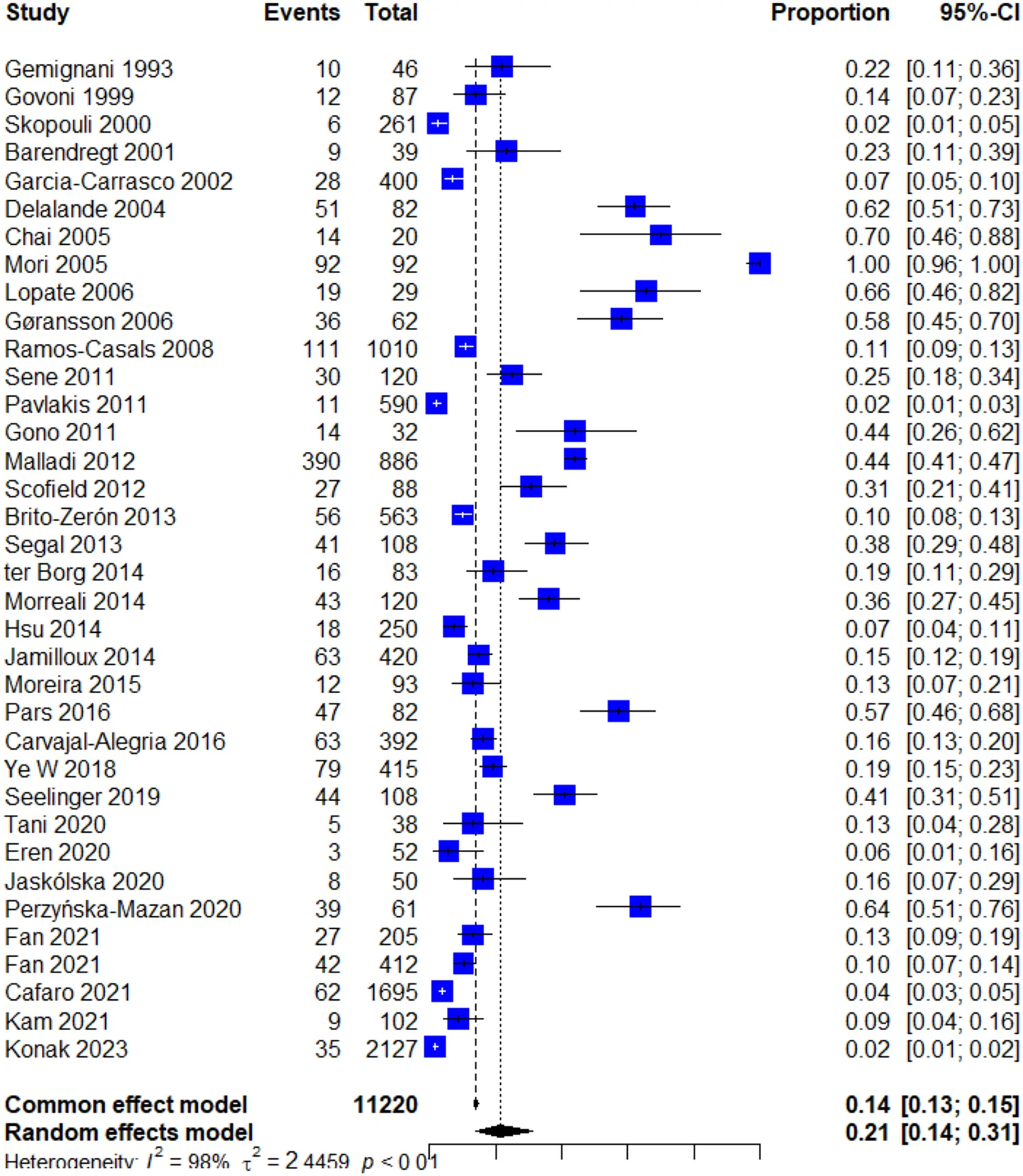
Meta-analysis of the central nervous system manifestations in patients with Sjogren’s disease

The original search yielded 977 results, 310 which matched the initial interest. After the abstract examination and further analysis of the remaining studies, we included 51 original reports (Fig. 1). Important topic sections summarize PNS and CNS aspects, and the authors formulated ten recommendations based on them. Figures 2 and 3 show pooled results from studies evaluating the proportion of nervous system manifestations in primary SjD and forest plots of the prevalence meta-analysis. Five critical studies among the 51 included predate the 2002 classification criteria. Regarding peripheric neurological manifestations, these affections were present in 21% of patients (95% CI 13–31%; 29 studies; 23,616 participants) (Fig. 2). Central nervous system manifestations were reported in 21% of the patients (95% CI 14–31%; 36 studies; 11,220 participants) (Fig. 3). In the systematic review we did not identify studies specifically analyzing accuracy for diagnosis or treatment of different neurological or psychiatric manifestations in SjD. Therefore, no meta-analysis could be done regarding these two aspects, so we present the results descriptively.

Given the broad spectrum of presentations, different pathogenic mechanisms respond to neurological injuries and symptoms [27, 28, 37–39]. Anatomically, there are three categories for didactic purposes: CNS, PNS, and autonomic nervous system [21]. PNS represents the largest group, followed by CNS and cranial neuropathy [21, 27, 40]. Commonly, they precede the diagnosis of SjD by a few years [10, 18, 41]. The use of the 2016 American College of Rheumatology (ACR)/European League Against Rheumatism (EULAR) classification criteria for SjD, in which extra glandular signs are inclusion criteria even in the absence of complaints of dryness, may contribute to an earlier diagnosis [34]. In these cases, performing a labial salivary gland biopsy becomes essential if there is no laboratory evidence of an immune-mediated disease to confirm the diagnosis.

The involvement of the neurological system modifies the SjD profile with higher rates of disease activity assessed by the ESSDAI [19] and more complaints measured by ESSPRI (the EULAR Sjögren’s Syndrome Patient Reported Index) and QoL instruments. In an analysis of a large Italian SjD cohort, axonal sensorimotor polyneuropathy (SMP) is linked to risk factors of lymphoproliferative disease (purpura, hypocomplementemia, and cryoglobulinemia). In contrast, individuals without neuropathy present a milder clinical and immunological profile [28]. How immunological and molecular profile drives SjD clinical phenotypes is still an evolving topic [29, 42]. The neurological evolution is variable, although it tends to be chronic, insidious, and debilitating despite treatment [43].

***Important topic***:

- *Neurological involvement of SjD is anatomically divided into three categories: central*,* peripheral*,* and autonomic nervous systems.*
- *The disease activity assessment tool*,* the EULAR Sjögren’s Syndrome Disease Activity Index (ESSDAI)*,* includes several manifestations; however*,* some neurological and psychiatric disorders are not evaluated within the instrument.*
- *Neurological manifestations may precede dry symptoms by a few years*,* making SjD diagnosis difficult.*
- *The neurological and psychiatric disorders attributable to SjD cover a vast and heterogeneous spectrum of manifestations*,* some of which are often not recognized as part of the underlying disease.*

***Recommendation***:

1. *It is recommended that the ESSDAI be used as an auxiliary tool for diagnosing and evaluating the activity of neurological disease during SjD*. **Level of Agreement: 100%**.
2. *It is recommended that SjD be investigated in patients with neurological syndromes*,* even in cases without dryness*,* mainly if there is no other apparent enlightenment for the diagnosis*. **Level of Agreement: 100%**.
3. *It is recommended to attend to neurological conditions in patients with evidence of autoimmunity (e.g.*,* antinuclear*,* anti-Ro/SSA*,* anti-La/SSB antibodies*,* cryoglobulins*,* rheumatoid factor)*,* presence of biomarkers (hypergammaglobulinemia*,* low levels of complement)*,* and in those with symptoms of dryness or a previous diagnosis of SjD*. **Level of Agreement: 100%**.
4. *It is recommended to manage patients with SjD and neurological disease at*,* or in close collaboration with*,* centers of expertise using a multidisciplinary approach*. **Level of Agreement: 100%**.

## Peripheral nervous system

Peripheral neuropathies (PN) include sensory, sensorimotor, and autonomic involvements. The most frequent PNs are sensory axonal polyneuropathy and small fiber neuropathy [16, 21, 41, 44]. In a retrospective study of 563 patients with primary SjD, 158/563 underwent nerve conduction studies [45]. Of these patients, 55/158 (35%) presented PN attributed to SjD (other causes ruled out), 24 (44%) had sensory motor axonal polyneuropathy; 15 (27%) had pure sensory polyneuropathy; 15 (27%), multiple mononeuropathies; and 1 (2%), demyelinating polyradiculoneuropathy [45].

The pathophysiology of PNS involvement in SjD is not entirely understood. Still, vasculitis of the vasa nervorum, necrotizing vasculitis, lymphocytic infiltration of the dorsal root ganglia causing ganglionitis, and the participation of autoantibodies directed to the muscarinic M3 receptors and antineuronal antibodies are possible mechanisms [9]. The initial investigation should consider the type of nerve involved (i.e., motor, sensory, or autonomic), fiber classification (i.e., small, myelinated, or unmyelinated), associated symptoms, neurophysiological tests, and the complete patient’s medical history (familial neuropathy, comorbidities, exposure to substances or medications). Combining clinical presentation, physical examination, serological results, electromyography, and, in some cases, nerve, muscle, or skin biopsies can more effectively differentiate each phenotype. Nowadays, nerve ultrasound is a practical, non-invasive, and efficient tool for detecting nerve damage of autoimmune origin, especially in demyelinating nerve damage patterns [46].

Older age, the presence of cryoglobulins, anti-neutrophil cytoplasmic antibodies with perinuclear pattern (p-ANCA), and beta-2 glycoprotein antibodies might carry a risk for SjD-related PN development [47, 48]. Major or minor B-cell activation (e.g., hypergammaglobulinemia, anti-Ro/SS-A, anti-La/SS-B, antinuclear antibodies (ANA) and rheumatoid factor) seems connected with specific sets of neuropathies, such as sensorimotor neuropathy or non-ataxic pure sensory neuropathy, respectively [44, 49].

***Pure sensory axonal polyneuropathy (PSP*)** is the most frequent form. It may often precede or coincide with the diagnosis of SjD. Sometimes, it is subclinical and, therefore, undiagnosed. Painful distal paresthesia is typically symmetric and length-dependent, affecting the distal lower extremities (burning feet), a common symptom. Physical examination demonstrates mild distal sensory deficits, including proprioception, light touch, and vibratory sensation Electrodiagnostic studies reveal reduced sensory nerve action potential and acute or chronic denervation features. The course of peripheral sensory axonal neuropathy is insidious and may progress slowly with ascending worsening in lower and upper limbs and motor impairment [21, 41, 44].

***Axonal sensorimotor polyneuropathy (SMP)*** may arise from the evolution of PSP, leading to the involvement of motor fibers. Patients experience tingling or numbness in affected areas, motor weakness, muscle stiffness, paresis of the toe, extensors of the feet, and foot drop as the most common presentation. Deep tendon reflexes become diminished or absent, particularly the Achilles tendon. A length-dependent sensory or sensorimotor neuropathy requires evaluation for differential diagnosis with other conditions (e.g., diabetes, sarcoidosis, paraproteinaemia, infection, neurotoxicity due to alcoholism, drugs, and toxins) [21, 41, 44]. Many adverse prognostic aspects, such as more aggressive disease (including purpura and other extra-glandular manifestations) and biomarkers (leukopenia, low complement, and cryoglobulinemia), characterize SjD patients with SMP [28].

***Small fiber neuropathy (SFN)*** occurs due to damage to Aδ small myelinated fibers or unmyelinated C fibers that conduct nociceptive stimuli and temperature. It is supposedly a common type of neuropathy in SjD, characterized by both positive (painful, burning, or prickly paraesthesias, allodynia, and hyperalgesia) and negative (hypoalgesia, hypoesthesia) sensory symptoms of chronic onset. Pain, the most important manifestation, is continuous regardless of the stimuli, worsens with rest, and impairs sleep. It can affect the trunk, proximal areas of limbs, face, and scalp in a non-dermatomal pattern. As a densely innervated tissue, the cornea may be involved, triggering additional ocular discomfort. Autonomic symptoms, comprising changes in sweating, visual accommodation, or bowel function, also occur [41, 50–53]. Neurological examination varies from normal to pain and thermal dysfunction in a non-length-dependent distribution. Epicritic and proprioceptive sensitivity is standard, as are deep tendon reflexes. Conventional electrophysiological methods show no abnormalities, as they only detect large fiber damage, the reason for suspicion and misdiagnosis of psychosomatic disorders [54–56].

Small fiber neuropathy has a poorly understood pathophysiology. Inflammatory mediators such as IL-1β and TNFα, which reduce the mechanical nociceptive threshold, were found in high concentrations in the skin of patients with SjD-related SFN [50]. Modulation of tryptophan catabolism induced by interferon-gamma (INF-γ) and mediated by indolamine-2,3-dioxygenase leads to neuroactive metabolites, and this deviation is another concurrent aspect in pain and behavior triggers [38, 57].

Diagnosing small fiber neuropathy (SFN) presents significant challenges, due to limited awareness among physicians and the requirement for specific diagnostic tests. Quantitative sensory testing, laser-evoked potentials, sympathetic skin response, electrochemical skin conductance, and histopathological studies are not routinely available [58, 59]. A punch skin biopsy with reduced intraepidermal nerve fiber density confirms the diagnosis [50, 60]. Confocal microscopy, a non-invasive and reproducible method for quantifying small nerve fiber degeneration and regeneration, can be useful for diagnosing and monitoring it [52]. The occurrence of SFN is not accurately determined. It is estimated that approximately 5–10% of individuals with SjD experience the condition [49, 61]. A previous analysis compared 23 patients with SjD and SFN to 98 SjD patients without SFN. A higher frequency of males was observed (30% vs. 9%, *p* < 0.01), along with decreased frequencies of polyclonal gammopathy (*p* < 0.01) and antibodies, specifically anti-Ro52 (*p* = 0.01), anti-Ro60 (*p* = 0.01), and RF (*p* < 0.01) in SjD with SFN [60].

***Sensory neuronopathy (ganglionopathy)*** is a rare injury to large fibers of the dorsal root ganglion, mediated by the infiltration of CD8 T lymphocytes and dendritic cells in the spinal cord. The process results in a multifocal pattern of sensory deficits. Humoral immune dysfunction plays a minor role. Paraesthesia, pain, upper limb and gait ataxia, difficulty with fine movements due to impaired proprioception, reduced vibration sense, areflexia, and Romberg sign are the key features *for diagnosis*. Au*tonomic dysfunction may occur*,* but muscle strength is typically maintained* [44, 62–64]. The association between ganglionopathy and other underlying conditions besides SjD, such as celiac disease, neurotropic viral infections, and cancer (with anti-Hu antibody positivity), is also mentioned [64]. Electrodiagnostic studies show multifocal involvement with decreased or absent sensory nerve action potential and no motor nerve conduction abnormalities, contrasting the usual length-dependent pattern found in axonal neuropathies [65, 66]. Magnetic resonance imaging (MRI) shows a T2-weighted hyperintense lesion at posterior columns and volumetric reduction in the cervical area resulting from dorsal root degeneration of their projections in the gracile and cuneate fasciculi [66, 67]. Although the excisional biopsy of the dorsal root ganglion is the diagnostic gold standard, this practice is exceptional. Individuals with SjD and ataxic sensory neuronopathy tend to be older and have a lower frequency of anti-Ro/SS-A and anti-La-SSB antibodies than those unaffected. The evolution of the disease is insidious and debilitating [27, 44]. Currently, there are no randomized clinical trials on treatment strategies; the management approach relies on observations from a limited number of cases. Importantly, SjD is one of the most frequent causes of this rare neurological disorder [21, 68].

***Multiple mononeuropathies*** occur as a result of necrotizing vasculitis of the *vasa nervorum*, T cell and macrophage infiltration, and ischemia-induced nerve damage. The primary clinical features include sensory and motor deficits in the affected regions, asymmetry, painful paresthesias, and a rapid progression of symptoms. Longer nerves are affected first, so foot and wrist drops are commonly encountered manifestations. Electrophysiological studies reveal axonal damage and pseudoblocks in areas of nerve ischemia, and a nerve biopsy may be necessary for a definitive diagnosis. Concomitant signs of vasculitis in other organs, such as the kidneys and skin, are strongly linked to serum cryoglobulins. Purpura, glomerulonephritis, hypergammaglobulinemia, and the depletion of complement factor C4 are concurrent findings [21, 61].

***Chronic Inflammatory Demyelination Polyneuropathy (CIDP)*** is a rare condition. It develops with a progressive, symmetrical loss of muscle strength affecting proximal and distal parts of the limbs, sensory dysfunctions, and reduction of deep tendon reflexes. The electromyography and nerve conduction studies reveal demyelination and conduction abnormalities (increased distal motor latency, conduction block, and absence of F waves). Hyperproteinorraquia, evidence of loss of myelinated fibers in the nerve biopsy, and clinical improvement with immunotherapy are complementary findings that support immunologic pathophysiology involving both the humoral and the cellular responses. The available treatment is complex, with corticosteroids, plasmapheresis, and intravenous immunoglobulin (IVIg) identified as the most feasible alternatives. Immunosuppressive regimens are reserved for failure of the initial therapy [43].

### Cranial neuropathy

Cranial nerves can be affected in their central or peripheral course. The main pairs involved are V, VII, and VIII, independently from each other and in descending order of frequency. In two series of SjD patients with neurological involvement, the frequency of peripheral cranial neuropathies ranged from 16 to 20%, with the trigeminal being most commonly affected [16, 41]. A prosaic clinical form is the unilateral lesion of the maxillary branch of the trigeminal nerve or trigeminal ganglion, causing sudden, intense facial pain. The second most frequent condition is peripheral facial paralysis, an asymmetric disturbance of mimicry and motricity of the face, and secretory dysfunction of the lacrimal and salivary glands. Then, hearing impairment and vestibular symptoms, such as vertigo, tinnitus, and ear fullness, are more common than often recognized [16, 41, 61]. High-frequency sensorineural hearing loss happens in up to 25% of patients with SjD. However, it is not attributed to SjD partly because of its natural incidence in aging, partly because it is often oligosymptomatic [69–71]. Several combinations between pairs and multiple involvements may occur [16, 41]. The immunologic mechanisms presume antibody-dependent cell-mediated cytotoxicity to local antigens with the activation of the complement system; FcR-mediated inflammatory reaction by immune complex deposition; micro vasculitis, anticardiolipin antibodies and ischemia [72]; local immune response by resident macrophages and oxidative stress mechanism [8, 9, 73, 74]. The treatment involves administering high doses of corticosteroids. Based on the apparent pathogenic mechanisms, some studies have considered the effects of immunosuppressive drugs such as methotrexate or cyclophosphamide, infliximab, and anecdotal cases showed responsiveness to heparin and plasmapheresis [72, 75, 76].

### Autonomous nervous system

Disorders of the autonomic nervous system and dry symptoms critically affect the quality of life of SjD patients [77]. Dysautonomia may occur allied to other forms of neuropathies, such as small fiber neuropathy and ganglionopathy [51]. Adie’s pupils, accommodation disturbance, disorders of gastrointestinal, sexual and bladder activity, orthostatic hypotension, heart arrhythmia, secretomotor and temperature control dysfunction, and anhidrosis are some examples. Electrophysiological methods, such as microneurography, sympathetic skin response, quantitative sudomotor axon reflex test, orthostatic test, and heart rate variability measurement, are valuable for examining the autonomic nervous system. The actual occurrence of dysautonomia in SjD patients is uncertain because of the diverse definitions used for classification and the unavailability of assessment tests. Additionally, the function of the autonomic system is modulated by emotional states, environmental conditions, and the consumption of stimulants and drugs [78].

The causes for the development of dysautonomia in SjD include cytokines interfering with cholinergic neurotransmission, the action of antibodies directed to the muscarinic receptors, destruction of autonomic nerve fibers, and inflammatory T lymphocytic infiltration in the area of root ganglia and nerves of the autonomic nervous system [73]. Despite all the distress of the disease, autonomic neuropathy treatment is only supportive.

***Important topic***:

- *Peripheral neurological manifestations are often associated with constitutional and visceral symptoms and alteration of serum biomarkers. These conditions may correlate with higher rates of disease activity as assessed by the ESSDAI*,* although many potential manifestations are not contemplated in this instrument.*
- *Peripheral SN involvement is more common than CNS involvement*,* and some of them can lead to poorer QoL and an increased risk of lymphoma.*
- *Survival seems reduced in patients with peripheral neuropathy*,* especially in those with mononeuritis multiplex.*
- *Neurological manifestations in SjD require special attention for the differential diagnosis with infectious (leprosy and viral infections by HCV*,* HIV*,* HTLV)*,* metabolic (diabetes mellitus)*,* hereditary diseases (Fabry disease*,* amyloidosis)*,* paraproteinemia and neoplasms*,* B12 and selenium deficiency*,* alcohol or drug toxicity*,* as well as the coexistence of other inflammatory diseases of autoimmune nature (celiac disease*,* myasthenia gravis*,* and Hashimoto’s disease).*
- *Some drugs involved in peripheral neurologic symptoms are antibiotics (isoniazid*,* metronidazole*,* ethambutol*,* nitrofurantoin*,* colistin*,* dapsone); antimitotics (vincristine*,* cisplatin*,* paclitaxel*,* vinblastine*,* doxorubicin); antivirals (DDI*,* DDC*,* alpha-interferon); and others (thalidomide*,* colchicine*,* gold salts*,* penicillamine*,* chloroquine*,* cyclosporine*,* amiodarone*,* phenytoin*,* disulfiram*,* lithium*,* cimetidine).*

***Recommendation***:

(5) *It is recommended that every patient be accurately assessed for neurological signs and symptoms at diagnosis and any complaints during follow-up visits*. **Level of Agreement: 100%**.
(6) *It is recommended that the most comprehensive assessment for patients with SjD and neurological complaints be performed*,* starting with a complete neurological examination. Appropriate complementary tests should be applied following syndromic and topographical clinical diagnoses*. **Level of Agreement: 100%**.
(7) *It is recommended that patients with SjD and peripheral neurological impairment*,* due to the increased risk of lymphoma*,* be evaluated for serum biomarkers (cytopenias*,* cryoglobulins*,* C4 fraction of complement*,* gammaglobulinemia)*,* for enlargement of salivary glands*,* lymphadenopathy*,* and constitutional symptoms (fever*,* night sweats*,* unintentional weight loss)*. **Level of Agreement: 100%**.

## Central nervous system

Involvement of the CNS may be severe and promote high morbidity and mortality, functional loss, and defective response to treatment. It is challenging to distinguish between the fortuitous association of SjD and another primary neurological disease from the neurological manifestation in the clinical spectrum of SjD, especially when the neurological condition precedes dry symptoms. Moreover, attributing CNS involvement to SjD requires careful evaluation to exclude infection, small vessel disease related to atherosclerosis, and adverse drug effects, among other causes. According to different authors, CNS disease is quite comprehensive and ranges from a headache or mild cognitive dysfunction to severe conditions, such as meningitis or seizures [13, 14, 16, 21]. Didactically, the classification into focal and multifocal or diffuse involvement is the most frequent [21].

CNS involvement accounted for 5.8% of cases in a series of 424 patients with SjD and was the initial manifestation in 52% [25]. In another series comprising 56 patients with SjD and CNS involvement, focal and multifocal brain and/or spinal cord syndromes such as optic neuritis (23%), multiple sclerosis-like syndromes (18%), and primary multiple sclerosis (23%) were described, along with diffuse involvement such as encephalopathy (2.4%) and cognitive disorders (11%) [16]. The pathophysiology of CNS disease in SjD is unknown, but lymphocytic infiltration of tissues, small vessel vasculitis leading to ischemia, and the action of antineuronal antibodies may occur [8, 9].

Focal or multifocal involvement of the brain and spinal cord as a result of vasculitis or demyelination may manifest as stroke, transient ischemic accident, hemorrhage, motor and sensory deficits, aphasia, seizures, pseudotumoral lesions, pure sensory or sensorimotor deficit syndrome similar to multiple sclerosis (MS-like), transverse myelitis, and optic neuropathy.

SjD-related MS-like syndromes affect some brain areas, such as the hypothalamus, periventricular region, and brainstem. The spinal cord involvement can range from transverse myelitis to progressive myelopathy [79, 80]. Oligoclonal bands in the cerebrospinal fluid (CSF) and typical MRI imaging contribute to the diagnosis. In the meantime, non-specific white matter lesions are usually more frequent in SjD and correlate with age, ischemic microangiopathy, and cardiovascular risk, as in control subjects [81]. Thus, they tend to spare the subcortical U-fibers of the corpus callosum and spinal cord and better affect the basal ganglia with lacunae formation [82]. Additional immunological mechanisms rather than vasculopathy are required to explain demyelination. The vascular injury appears associated with antineuronal and anti-Ro/SS-A antibodies [82].

Some features assist the diagnostic process: a- anti-Ro/SS-A and anti-La/SS-B antibodies are common in SjD and absent in most cases of MS; b- oligoclonal bands in the cerebrospinal fluid are present in more than 90% of MS cases, and only 20% in SjD (the finding of three or more bands is rare in SjD); c- the brain MRI shows hyperintense areas on T2 and FLAIR in the subcortical and periventricular white matter, cerebral cortex, basal ganglia, and corpus callosum in MS (as opposed to SjD); d- MRI juxtacortical and periventricular hypointense T1-weighted ovoid lesions (“black holes”) are characteristic of MS; e- the spinal cord injuries are commonly restricted to ≤ 2 vertebral segments and present partial transverse involvement of the spinal cord in MS, unlike SjD [8, 9]. Further systemic manifestations, such as arthritis and interstitial lung disease, also contribute to the suspicion of SjD [61].

Demyelinating disease in SjD sometimes has neuromyelitis optica (NMO) aspects, with recurrency of myelitis and optic neuritis episodes associated with anti-aquaporin-4 (AQP4) IgG antibodies. It is controversial whether this condition is a manifestation of SjD or an overlap of SjD with neuromyelitis optic spectrum disorder (NMOSD). This association varies from 7 to 31.8% in the studies depending on the presence of AQP4 antibodies in CSF [80, 83, 84], and their coexistence possibly modulates the clinical expression of each disease. Interestingly, no association of AQP4 antibody with CNS manifestation other than NMOSD has been demonstrated [83].

Vascular disease, *transient ischemic attack*,* and* stroke in SjD patients without traditional cardiovascular risk factors have been rarely described [9], so vasculitis or associated antiphospholipid syndrome should be considered in these cases [85].

### Diffuse encephalic involvement

Dementia, psychiatric and sleep disorders, aseptic lymphocytic meningitis, and meningoencephalitis are examples of diffuse involvement. Patients with SjD have an incremental risk for anxiety and depression when compared to the general population [86], poorer sleep, and a worse quality of life [86, 87]. Headache is a frequent symptom, but it occurs similarly in healthy controls, and there is no definite association with fatigue, depression, serum autoantibodies, or findings on CSF or MRI [88]. In contrast, cognitive dysfunctions related to attention, processing speed, recent memory, and visuospatial performance have a multifactorial etiology involving pain, fatigue, depression, and possibly an immune-mediated mechanism [89–91].

**Aseptic meningitis (AM) and meningoencephalitis (AME)** generally develop in the context of still unknown SjD and disfavor infection. The disorders result from an autoimmune inflammatory process and present with pleocytosis in the cerebrospinal fluid (CSF), negative bacterial cultures, and negative virus amplification by polymerase chain reaction tests. AM and AME have a self-limiting course with spontaneous or corticosteroid-induced improvement. Initial symptoms include headache, fever, nausea, vomiting, disturbance of consciousness, and neck pain or rigidity. T2 fluid-attenuated inversion recovery sequence on gadolinium-enhanced MRI of the brain reveals diffuse enhancement of the meninges. In many cases, patients experience relapses, requiring glucocorticoid or immunosuppressive therapy [92].

### Cognitive disorders

Cognitive impairments range on a scale of severity from “mild”, which might be noticed by the patient or healthcare providers, to “severe” such as dementia that affects activities of daily living or disallows patients to function without assistance. As in SLE, antibodies directed against *N*-methyl-D-aspartate receptor subtype NR2 (anti-NR2) may interfere with memory, learning function, and mood [93]. Despite this, the relationship between anti-NR2 and cognitive dysfunction is still controversial and represents only one of the pathogenic mechanisms for cognitive impairments and mood disorders in SjD [37, 94].

Complaints of impaired memory, attention, concentration, and a feeling of “brain fog” are common. Neuropsychological testing detected a certain degree of cognitive dysfunction in almost 80% of patients, and in half of these, the disturbance was moderate to severe [89]. Dementia is rare and only occasionally reported [21, 95]. Brain MRI ranges from standard to findings of white matter changes and brain or hippocampal atrophy [21, 61, 95]. Possible reversible causes such as depression, anxiety, non-restorative sleep, chronic pain, hypothyroidism, vitamin deficiencies, and adverse drug effects should be investigated [21, 61, 95].

### Psychiatric disorders

Emotional deregulation is associated with changes in cognition and impaired social interaction [86, 91]. The most common abnormalities include depression and anxiety, while psychosis and obsessive-compulsive disorder are erratic. There is a lack of data on many aspects of psychiatric involvement. Patients submitted to psychiatric, neuropsychological, and neuroimaging examinations present neurobehavioral and neuroimaging alterations compared to controls [37, 94]. The detection of anti-NR2 antibodies in CSF and reduced hippocampal volume on brain imaging need further interpretation [37, 94]. To date, there is no unequivocal demonstration of the association between psychiatric disorders and serum autoantibodies. Abnormal electroencephalograms occur in about 33–48% of patients with CNS-SjD [96]. Brain MRI abnormalities, including non-enhancing T2 hyperintensities in the periventricular and subcortical areas, have been seen in up to 75% of adults with SjD and neuropsychiatric symptoms and 9% of adults with SjD without neuropsychiatric symptoms [97].

INF-γ-induced tryptophan catabolism implicated in psychiatric, inflammatory, and neurotoxic mechanisms is an emerging issue. Different expressions of type-I and -II IFN-γ may lead to various clinical phenotypes in SjD, one of them limited to widespread pain, dryness, and depression [29]. The kynurenine metabolic pathway (KYN) generates compounds with cytotoxic and neuroactive or cytoprotective actions. Kynurenic acid participates in tolerance and immunomodulation processes (induction of T-reg lymphocytes and dendritic cells with immunosuppressive action and glutamate receptor blockade). Hydroxykynurenine stimulates the apoptosis of neurons. Quinurinic acid is an NMDA receptor agonist [39]. According to some authors, the process hypothetically participates in the “wind-up” phenomenon of spinal cord posterior horn sensitization, hyperalgesia, allodynia, visceral pain, fatigue, exercise intolerance, hippocampal and trigeminal ganglion atrophy, depressive symptoms, cognitive impairment and dryness [38, 98]. The proposition sheds light on poorly clarified subjects, such as the dissociation between symptomatology and objective findings.

***Important topic***:

- *The CNS diseases in SjD are diverse*,* and CNS imaging and CSF analysis are important tools for diagnosis and differential diagnosis.*
- *Such involvements can be didactically classified into focal/multifocal involvements*,* such as stroke*,* optic neuritis*,* multiple sclerosis-like*,* and pseudotumoral lesions)*,* and diffuse involvements (aseptic meningoencephalitis*,* cognitive dysfunction*,* and psychiatric disorders).*
- *CNS involvement may cause significant functional loss and higher morbidity and mortality.*
- *Cognitive disorders*,* depression*,* encephalopathy*,* and meningitis are characteristic of diffuse involvement*,* which is more frequent and less severe. Vasculitis*,* demyelinating diseases with myelitis*,* optic neuritis*,* and motor neuron injury characterize focal involvement*,* which is rarer and life-threatening.*
- *The main risk factor for neurological manifestation in SjD patients is the previous occurrence of another neurological involvement.*

## Complementary diagnostic evaluation and management

Besides a complete medical history and physical exam, the type and topography of each neurological syndrome guide additional tests for the complementary evaluation. Worldwide, leprosy and diabetes mellitus remain the most frequent causes of peripheral neuropathy, making it essential to exclude these diagnoses. Other conditions in the SjD spectrum, such as the acid-base and electrolyte disturbances in tubulointerstitial nephritis, alterations of voltage-gated sodium ion channels (channelopathies), and myositis, also mimic neuropathic manifestations. Blood tests can detect vitamin deficiencies, abnormal immune function, thyroid disease, electrolyte disturbances, and myopathies [4, 11, 21].

Electromyography (EMG) is indicated chiefly for peripheral neuropathies. In cases where small fiber neuropathy and/or dysautonomia are strongly suspected, specialized tests may be included, such as an autonomic reflex screen and sensory tests for touch, vibration, temperature, and sweat [59, 99].

Nerve and skin biopsies are relevant diagnostic tools. Nerve histology and immunofluorescence staining may point to autoantibodies against paranodal proteins, reveal familial amyloid neuropathy, and is the gold standard for diagnosing peripheral nerve vasculitis. Modern imaging methods allow localization of nerve damage, such that targeted fascicular biopsies can be done [100]. Although not routinely available, a skin biopsy can differentiate length-dependent from non-length-dependent neuropathies and identify small fiber involvement [100]. A simple punch biopsy can determine intraepidermal nerve fiber density impairment and become a routine method to diagnose SFN [50, 60].

As mentioned, imaging tests of the brain and neuraxis are valuable in focal, diffuse, vascular, and demyelinating central nervous system lesions. The principal MRI findings are nonspecific white matter lesions of vascular origin in up to 80% of patients, commonly disconnected from the clinical presentation [101–103]. Single-photon emission computed tomography (SPECT) is valuable for assessing areas of compromised cerebral perfusion, mainly when analyzed in conjunction with MRI [80–82].

Patients presenting with optical neuritis or transverse myelitis should have AQP-4 and anti-myelin oligodendrocyte protein (MOG) antibodies tested, as multiple case reports are found in the literature of NMOSD occurring with coexisting SjD [83, 104]. These autoantibodies are essential for prognosis, guiding the chances of relapse and determining the type and duration of immunotherapy [105–107]. Overall, the peripheral and central nervous systems are affected differently in seropositive and seronegative anti-Ro and anti-La SjD patients [108–110]. In a study by Lauvsnes et al., anti-NR-2 antibodies were detected in serum (20%) and cerebrospinal fluid (12%) of SjD patients and appeared to be associated with memory dysfunction and depression [37, 107].

Older patients presented an association between neurological disease, glandular atrophy, and ANA positivity. In contrast, patients under 50 presented an association with serum complement consumption [20, 111].

Studies analyzing cerebrospinal fluid showed that most patients had no evidence of significant inflammation and that the test helped exclude infections and neoplasms [40]. Mild pleocytosis was identified in 9% of patients with peripheral neuropathy, 20% of patients with cranial neuropathy, and 25% of patients with CNS manifestations. Oligoclonal bands indicating intrathecal synthesis of IgG were observed in 26% of patients with peripheral neuropathy, 20% with cranial neuropathy, and 25% with central nervous system manifestations [40].

***Recommendation***

(8) *It is recommended to perform complementary exams to refine the syndromic and topographical aspects of neurologic disease and avoid misdiagnosis*. **Level of Agreement: 100%**.

I. *Cerebrospinal fluid is essential for the differential diagnosis of infections and CNS neoplasms. The results are within the reference range in most cases of neuropsychiatric SjD. Proteinorrhachia*,* lymphocytic pleocytosis*,* and oligoclonal bands are nonspecific findings in up to ¼ of patients with central*,* cranial*,* or peripheral involvement.*
II. *Imaging tests such as MRI of the brain and neuroaxis are helpful in the detection of focal and diffuse*,* vascular*,* and demyelinating lesions. The most frequent findings are lesions of vascular origin in the white matter*,* not specific*,* in up to 80% of the exams of symptomatic or non-symptomatic individuals.*
III. *SPECT (Single-photon emission computed tomography) is valuable*,* although non-specific*,* in assessing the perfusion of compromised brain areas when analyzed in conjunction with MRI.*
IV. *Electromyography* (*EMG*) and *nerve conduction studies (NCS) are important tools for diagnostic and topographic validation*,* particularly for peripheral neuropathies*,* and should be performed in addition to neurological physical examination.*
V. *Biopsy and histopathologic studies of nerve*,* dorsal ganglion*,* muscles*,* and skin are useful in revealing vasculitis*,* necrosis of the vasa nervorum*,* inflammatory T-cell infiltrate*,* and a reduction in the density of unmyelinated fibers in the epidermis*,* respectively.*
VI. *It is recommended that all patients with optic neuritis or myelitis be screened for anti-aquaporin 4 antibodies.*

(9) *It is recommended to perform early screening and periodic follow-up of patients with SjD and complaints of hearing loss*,* with or without cognitive impairment*,* investigating sensorineural hearing loss by pure tone audiometry*. **Level of Agreement: 100%**.

## Treatment

There is currently no approved treatment for Sjögren’s disease. Historically, the management has relied on expert opinion and the off-label use of effective drugs in comparable diseases, such as systemic lupus erythematosus and rheumatoid arthritis. The difficulty in developing effective therapies stems from differences in the pathogenetic basis of each species of involvement. Common molecular signatures among patient subgroups could facilitate the development of targeted therapeutics in the future. The presenting symptoms and signs dictate the treatment of neuropathies, varying from symptomatic management to immunosuppressive therapy when they are rapidly progressing, life-threatening, or include severe motor deficits. Early initiation of supportive care, such as pain and psychiatric symptoms management and physical and occupational therapy, may minimize morbidity and be instituted regardless of the immunosuppressive regimen [41]. The principal set of recommendations on the management of SjD, formulated by the EULAR, still has several unmet needs requiring additional research [112]. However, a systematic review of the treatment of neurological conditions was not within the scope of this study.

## Peripheral nervous system management

For patients with mild, stable disease related to sensory and sensorimotor axonal neuropathy, the opening treatment may be just symptomatic management [12, 113, 114]. For a severe sensory disease that is not relieved by conventional treatments or progresses with motor impairment, IVIg at a dose of 2 g/kg/monthly has been successfully reported [115–117]. Monthly pulses of methylprednisolone and cyclophosphamide are second-line options reserved for severe refractory cases [112]. Rituximab and disease-modifying agents, such as mycophenolate mofetil (MMF) and azathioprine, have been evaluated with often disappointing results [116]. It is noteworthy that all proposed schemes derive from the observation of a limited series of patients.

Treatment for small fiber neuropathy focuses on pain relief. First-line therapeutic agents include gabapentin and pregabalin. Serotonin-norepinephrine reuptake inhibitors (such as duloxetine or venlafaxine) may replace or be added to first-line therapy if pain control is insufficient [113]. Tricyclic antidepressants are effective but may exacerbate the existing sicca component, whereas opioid analgesics have questionable efficacy. Experimental immunomodulatory or immunosuppressive therapy may help patients who have failed symptomatic therapy. Similarly, IV Ig may be used based on beneficial results in anecdotal case reports, although the duration of treatment remains unclear [117, 118]. Corticosteroid therapy and rituximab trials provided unsatisfactory responses [41, 119]. The treatment of SFN relies on a multimodal concept and includes causative, pathophysiologic, and symptomatic measures. It depends on clinical presentation and should be individualized to alleviate pain and optimize functionality [120].

Immunosuppressive drugs, intravenous immunoglobulin, anti-TNF, anti-CD20 therapies, and corticosteroids are all controversial therapeutic modalities for managing sensory ataxic neuronopathy [64, 121]. In several case reports and reviews, the corticosteroid is the most widely prescribed, and IV Ig is often used together as the first line of treatment [41, 64, 68, 122, 123]. Studies with immunotherapy (such as rituximab, infliximab, interferon alpha), synthetic drugs (such as hydroxychloroquine, cyclophosphamide, tacrolimus, azathioprine, d-penicillamine, MMF), and plasmapheresis have conflicting responses, perhaps due to the typical delay in introducing therapy [68, 121, 122, 124, 125].

Mononeuritis multiplex is usually treated with cyclophosphamide and high-dose corticosteroids, which are usually largely effective [16, 112, 126]. Rituximab can be added to treat cryoglobulinemic vasculitis-related PNS [83, 119].

Cranial neuropathy and multiple cranial neuropathies related to vasculitis, except for isolated trigeminal neuralgia, are treated with corticosteroids [41]. Radiculoneuropathy may respond to IV Ig, as demonstrated in the small series [66]. Chronic inflammatory demyelinating polyneuropathy should be treated with corticosteroids, plasmapheresis, and IVIg [127]. Autonomic neuropathy receives supportive care only since a series of patients treated with IV Ig and prednisone have demonstrated no favorable outcomes [41].

## Central nervous system management

The relative rarity of CNS manifestations has not permitted clinical trials. Thus, treatment recommendations are based on expert opinion and case series, so pharmacologic therapy is mainly empiric. In general, the first line for severe, acute, or rapidly progressive forms of CNS involvement such as demyelinating disease, cerebral vasculitis, and meningoencephalitis is a high-dose of corticosteroid (intravenous methylprednisolone) and monthly cyclophosphamide, followed by prednisone (1 mg/kg/day) [16, 112, 121, 128]. As in other critical manifestations of autoimmune diseases treated with a similar approach, the objective is to gradually taper prednisone doses within 2–3 months, targeting 5 mg/daily or less. No studies detail the success rates of this protocol in SjD CNS disease. Alternative therapy and remission maintenance include drugs such as azathioprine and mycophenolate mofetil, but the absence of head-to-head studies comparing the efficacy and safety profile of immunosuppressive agents does not certify a recommendation on the use of one agent over another [129, 130]. The benefits of rituximab have been unimpressive in small case series [131], although it attends the rescue treatment of cryoglobulin-associated vasculitis and NMOSD, with or without plasma exchange support [112, 129, 130]. The cases of EM-like should receive specific EM therapies [112]. For psychiatric involvement or indolent signs such as mental fog, monitoring with supportive treatment is proper. Immunosuppression in SjD patients with dementia is only acceptable after excluding more common causes of dementia [121, 132].

***Important topic***:

- *The treatment of neurological manifestations is empiric*,* based on evidence acquired from small case series in uncontrolled studies and guided by expert opinion*,* extrapolated from treating other extra glandular manifestations of SjD itself or treating similar manifestations in other autoimmune diseases.*
- *Synthetic immunosuppressive drugs are used mainly as glucocorticoid-sparing agents*,* with little evidence supporting the choice of one agent over another. The lack of studies comparing its efficacy in SjD does not allow a specific recommendation*,* except about safety profile*,* considering the characteristics and comorbidities of the patients.*
- *There is no information available about the dose*,* route of administration*,* and length of treatment.*

***Recommendations***:

- *It is recommended that the pharmacological management of the neurologic manifestations focus on the pathogenic mechanism*,* which differs depending on the type of involvement*. **Level of Agreement: 100%**.

## Conclusion

The assessment of systemic involvement in SjD is not adequately incorporated into the health care routine. This guideline produced by the Brazilian Society of Rheumatology focuses on the forms of neurological, cognitive, and psychiatric impairment present in primary Sjögren’s disease. The best evidence on pathophysiology and diagnostic evaluation available to date was compiled. The proposals are an initial step toward developing an optimal diagnostic approach. We provide 10 recommendations with a high level of agreement among experts. Due to the heterogeneous status of scientific evidence, we suggest attentiveness and a case-by-case evaluation following these recommendations in clinical practice.

## Electronic supplementary material

Below is the link to the electronic supplementary material.

Supplementary Material 1

Supplementary Material 2

## Data Availability

No datasets were generated or analysed during the current study.
