# Supplementary material for: Recommendations on neurologic, cognitive, and psychiatric manifestations in patients with Sjögren’s disease by the Brazilian Society of Rheumatology
Source: Adv Rheumatol. 2025 Feb 11;65:7. doi: 10.1186/s42358-025-00438-7 (PMC13488763; doi:10.1186/s42358-025-00438-7)
Supplement: Supplementary file 1 [file 42358_2025_438_MOESM1_ESM.docx]

Embase via Elsevier

#1.

'Sjoegren syndrome'/exp OR 'dacryosialoadenopathia atrophicans' OR 'dyssecretosis, mucoserous' OR 'Gougerot Houwer Sjoegren syndrome' OR 'Gougerot Mulock Houwer Sjoegren syndrome' OR 'Gougerot Sjoegren disease' OR 'Gougerot Sjoegren syndrome' OR 'Gougerot Sjogren disease' OR 'Gougerot Sjogren syndrome' OR 'Gougerot-Sjogren syndrome' OR 'Mikulicz Gougerot Sjoegren syndrome' OR 'Mikulicz Radecki syndrome' OR 'mucoserous dyssecretosis' OR 'Mukilicz Radecki syndrome' OR 'oculobuccopharyngeal dryness' OR 'rheumatic sialosis' OR 'sialosis, rheumatic' OR 'sicca syndrome' OR 'Sjoegren disease' OR 'Sjoegren`s syndrome' OR 'Sjogren disease' OR 'Sjogren syndrome' OR 'Sjogren`s syndrome' OR 'xerodermosteosis' OR 'Sjoegren syndrome'

#2.

'neurologic disease'/exp OR 'nervous disease' OR 'nervous disorder' OR 'nervous system disease' OR 'nervous system diseases' OR 'nervous system disorder' OR 'neural disease' OR 'neurogenic disease' OR 'neurologic complaint' OR 'neurologic disorder' OR 'neurologic disturbance' OR 'neurologic dysfunction' OR 'neurologic manifestations' OR 'neurologic sign' OR 'neurologic symptom' OR 'neurologic syndrome' OR 'neurological complaint' OR 'neurological deficiency' OR 'neurological disease' OR 'neurological disorder' OR 'neurological disturbance' OR 'neurological sign' OR 'neurological symptom' OR 'neurological syndrome' OR 'sign, neurologic' OR 'symptom, neurological' OR 'neurologic disease'

#3.

#1 AND #2

#4.

#3 AND [embase]/lim NOT ([embase]/lim AND [medline]/lim)

Portal Regional BVS

#1.

MH:"S ndrome de Sjogren" OR (S ndrome de Sjogren) OR (Sjogren's Syndrome) OR (S ndrome de Sj gren) OR (S ndrome Seca) OR (S ndrome Sicca) OR MH:C05.550.114.154.774$ OR MH:C05.799.114.774$ OR MH:C07.465.815.929.669$ OR MH:C11.496.260.719$ OR MH:C17.300.775.099.774$ OR MH:C20.111.199.774$

#2.

MH:"Manifesta es Neurol gicas" OR (Manifesta es Neurol gicas) OR (Neurologic Manifestations) OR (Manifestaciones Neurol gicas) OR (Deficits Neurol gicos) OR (Defici ncias Neurol gicas) OR (Sinais e Sintomas Neurol gicos) OR MH:C10.597$ OR MH:C23.888.592$

#3.

#1 AND #2

CENTRAL via Cochrane Library

#1.

MeSH descriptor: [Sjogren's Syndrome] explode all trees

#2.

(Sjogrens Syndrome) OR (Syndrome, Sjogren's) OR (Sjogren Syndrome) OR (Sicca Syndrome) OR (Syndrome, Sicca)

#3.

#1 OR #2

#4.

MeSH descriptor: [Neurologic Manifestations] explode all trees

#5.

"Manifestation, Neurologic" OR "Neurological Manifestations" OR "Neurologic Manifestation" OR "Neurologic Signs and Symptoms" OR "Manifestations, Neurologic" OR "Manifestations, Neurological" OR "Manifestation, Neurological" OR "Neurological Manifestation" OR "Neurologic Deficits" OR "Deficit, Neurologic" OR "Deficits, Neurologic" OR "Neurologic Deficit" OR "Neurologic Symptoms" OR "Neurologic Symptom" OR "Symptom, Neurologic" OR "Symptoms, Neurologic" OR "Neurologic Findings" OR "Finding, Neurologic" OR "Findings, Neurologic" OR "Neurologic Finding" OR "Neurologic Signs" OR "Neurologic Sign" OR "Sign, Neurologic" OR "Signs, Neurologic" OR "Focal Neurologic Deficits" OR "Deficit, Focal Neurologic" OR "Deficits, Focal Neurologic" OR "Focal Neurologic Deficit" OR "Neurologic Deficit, Focal" OR "Neurologic Deficits, Focal" OR "Neurologic Dysfunction" OR "Dysfunction, Neurologic" OR "Dysfunctions, Neurologic" OR "Neurologic Dysfunctions"

#6.

#4 OR #5

#7.

#3 AND #6

Pubmed

(("Sjogren's Syndrome"[Mesh] OR "Sjogrens Syndrome" OR "Syndrome, Sjogren's" OR "Sjogren Syndrome" OR "Sicca Syndrome" OR "Syndrome, Sicca")) AND ("Neurologic Manifestations"[Mesh] OR "Manifestation, Neurologic" OR "Neurological Manifestations" OR "Neurologic Manifestation" OR "Neurologic Signs and Symptoms" OR "Manifestations, Neurologic" OR "Manifestations, Neurological" OR "Manifestation, Neurological" OR "Neurological Manifestation" OR "Neurologic Deficits" OR "Deficit, Neurologic" OR "Deficits, Neurologic" OR "Neurologic Deficit" OR "Neurologic Symptoms" OR "Neurologic Symptom" OR "Symptom, Neurologic" OR "Symptoms, Neurologic" OR "Neurologic Findings" OR "Finding, Neurologic" OR "Findings, Neurologic" OR "Neurologic Finding" OR "Neurologic Signs" OR "Neurologic Sign" OR "Sign, Neurologic" OR "Signs, Neurologic" OR "Focal Neurologic Deficits" OR "Deficit, Focal Neurologic" OR "Deficits, Focal Neurologic" OR "Focal Neurologic Deficit" OR "Neurologic Deficit, Focal" OR "Neurologic Deficits, Focal" OR "Neurologic Dysfunction" OR "Dysfunction, Neurologic" OR "Dysfunctions, Neurologic" OR "Neurologic Dysfunctions")
