# Supplementary material for: Recommendations on neurologic, cognitive, and psychiatric manifestations in patients with Sjögren’s disease by the Brazilian Society of Rheumatology
Source: Adv Rheumatol. 2025 Feb 11;65:7. doi: 10.1186/s42358-025-00438-7 (PMC13488763; doi:10.1186/s42358-025-00438-7)
Supplement: Supplementary file 2 [file 42358_2025_438_MOESM2_ESM.docx]

**Appendix 2**

**Appendix - Joanna Briggs Institute (JBI) Critical Appraisal Checklist**

| **Study ID** | **Was the sample frame appropriate to address the target**  **population?** | **Were study participants sampled in an appropriate way?** | **Was the sample size adequate?** | **Were the study subjects and the setting described in**  **detail?** | **Was the data analysis conducted with sufficient coverage**  **of the identified sample?** | **Were valid methods used for the identification of the**  **condition?** | **Was the condition measured in a standard, reliable way**  **for all participants?** | **Was there appropriate statistical analysis?** | **Was the response rate adequate, and if not, was the low**  **response rate managed appropriately?** |
| --- | --- | --- | --- | --- | --- | --- | --- | --- | --- |
| Gøransson LG 2006 | + | - | - | + | - | + | + | + | NA |
| Gökçay F 2008 | + | - | + | - | + | + | + | + | + |
| Akasbi M 2012 | + | + | - | + | + | + | + | + | NA |
| Birnbaum J 2016 | + | + | - | + | + | + | + | + | NA |
| Carvajal Alegria G 2016 | + | + | + | + | + | + | + | + | NA |
| Chai J 2005 | + | + | - | + | + | + | + | + | NA |
| Estiasari R 2012 | + | + | - | + | + | + | + | + | NA |
| Gono T 2011 | + | + | - | + | + | + | + | + | NA |
| Lauvsnes MB 2013 | + | + | + | + | + | + | + | + | NA |
| Lopate G 2006 | ? | ? | - | + | + | + | + | - | NA |
| Malladi AS 2012 | + | + | + | + | + | - | ? | + | + |
| Massara A 2010 | + | + | + | + | + | + | + | + | NA |
| Moreira I 2015 | + | + | + | + | + | + | + | + | NA |
| Mori K 2005 | + | + | + | + | + | + | + | + | NA |
| Morreale M 2014 | + | + | + | + | + | + | + | + | + |
| Ng W-F 2012 | + | + | - | - | - | + | + | + | NA |
| Pars K 2016 | + | + | - | - | + | + | + | + | NA |
| Qiao L 2015 | + | + | + | + | + | + | + | + | NA |
| Scofield AK 2012 | + | + | + | + | + | + | + | + | NA |
| Segal BM 2012 | ? | ? | + | - | + | + | + | + | + |
| Segal BM 2013 | + | + | + | + | + | + | + | + | + |
| Shen C-C 2015 | ? | + | + | + | + | ? | ? | + | NA |
| Tezcan ME 2016 | + | + | - | + | + | + | + | + | NA |
| Tjensvoll AB 2013 | + | + | + | + | + | + | + | + | NA |
| Barendregt PJ 2001 | + | + | - | + | + | + | + | + | NA |
| Ye W 2018 | + | + | + | + | + | + | + | + | NA |

+=Yes; -=No; ?=Unclear; NA=Not Applicable
